# Editorial: Transcription factors and regulation of transcriptional programs in fungi

**DOI:** 10.3389/ffunb.2022.1117910

**Published:** 2022-12-20

**Authors:** Richard B. Todd, Koon Ho Wong, Gustavo H. Goldman

**Affiliations:** ^1^ Department of Plant Pathology, Kansas State University, Manhattan, KS, United States; ^2^ Faculty of Health Sciences, University of Macau, Macau, Macau SAR, China; ^3^ Institute of Translational Medicine, Faculty of Health Sciences, University of Macau, Macau, Macau SAR, China; ^4^ Ministry of Education Frontiers Science Center for Precision Oncology, University of Macau, Macau, Macau SAR, China; ^5^ Faculdade de Ciências Farmacêuticas de Ribeirão Preto, Universidade de São Paulo, Ribeirão Preto, Brazil

**Keywords:** fungal transcription factors, transcriptional programs, systems biology, gene regulation, transcription activator, transcription repressor

Regulation of gene expression underlies the coordination of cellular processes. In fungi, transcriptional gene regulation is the major level of control. Transcriptional gene regulation is mediated by transcription factors (TFs) – transcription activator and repressor proteins – *via* interactions with promoters of the target genes they regulate, as well as interactions with other TFs, inducers, transcription regulators, chromatin modifiers and remodelers, co-factors, and the general transcription machinery. The importance of transcriptional control in fungi is evident in the wide expansion of TF gene families in Ascomycetes and Basidiomycetes for the regulation of transcriptional programs for development, metabolism, and response to environmental fluctuations (Todd et al., 2014). Historically, extensive studies of gene regulatory mechanisms in *Neurospora crassa, Aspergillus nidulans*, and *Saccharomyces cerevisiae* have been foundational to the understanding of TFs and their mechanisms of action. With the advent and increasing affordability of Next-Generation Sequencing technologies and their applications, increasing efforts are being made to understand TFs and genome-wide regulation of transcriptional programs in other fungi.

This Research Topic highlighted current research on fungal TFs, regulation of TF action in fungi, and the mechanisms whereby fungal TFs regulate the transcriptional programs that they control. Two articles highlight transcriptional controls of pathways for degradation of plant compounds, and two articles present analysis of TFs in an opportunistic human fungal pathogen.

Arentshorst et al.report the identification, in *Aspergillus niger*, of a conserved transcriptional activator-repressor module controlling the expression of genes involved in degradation of the plant compounds tannic acid and gallic acid. The transcriptional activator-repressor module consists of a Zn(II)2Cys6 transcription activator, TanR, and a repressor protein, TanX, encoded by an adjacent gene pair. The *tanR*Δ mutant is unable to utilize gallic acid and shows reduced utilization of tannic acid, whereas the *tanX*Δ mutant shows constitutive expression of the tannic acid degrading enzymes. The authors used transcriptomics analysis of the *tanX*Δ mutant compared with wild type to identify candidate gallic acid utilization genes, and used gene deletion analysis and phenotyping to identify a gene, *gacA*, encoding the likely first enzyme in the gallic acid catabolism pathway. The identification of this novel transcriptional activator-repressor module highlights a mechanistic theme common in transcriptional regulation in filamentous fungi, adding to the galacturonic acid utilization (GaaR/GaaX in *A. niger*) and quinic acid utilization (QutA/QutR in aspergilli, Qa-1F/Qa-1S in *N. crassa*) transcriptional activator-repressor regulatory modules (Geever et al., 1989; Lamb et al., 1990; Niu et al., 2017).

Kölle et al. present a comparative transcriptomics analysis of three fungi during brown rot decay. Two strains of *Rhodonia placenta* and one strain of *Gloeophyllum trabeum* – brown rot fungi used in wood durability testing – were assessed. A previous study had shown that brown rot wood degradation occurs in two steps (Zhang et al., 2016). Temporally distinct transcriptional programs were observed for the early oxidative phase and the subsequent enzymatic phase of wood degradation. The transcriptomes also demonstrated strategic differences between the three fungi. Furthermore, the responses to wood acetylation, a wood preservation treatment, were assessed, revealing delays in the transcriptional response. The authors performed hierarchical clustering of gene expression profiles to identify within their transcriptome data TFs that were affected by wood acetylation. This work demonstrated that brown rot occurs *via* a switch between transcriptional programs defining the two phases of degradation, and identified differences in the transcriptional programs that underlie alternative degradation strategies by different fungi.

Valero et al. focus on a novel *Aspergillus fumigatus* zinc finger TF, ZnfA, involved in calcium metabolism and tolerance to the antifungal echinocandin drug caspofungin. This study used chromatin-immunoprecipitation-sequencing (ChIP-seq) to reveal ZnfA target genes and uncover roles for ZnfA in iron regulation and cell wall organization in response to caspofungin treatment. Furthermore, analysis of double TF gene deletion mutants of *znfA*, with deletions of two other TF genes, *crzA* and *zipD*, revealed genetic interactions between these three TFs in the calcium and caspofungin response, suggesting a complex multi-factorial regulatory mechanism for calcium homeostasis and caspofungin resistance.

Silva et al. examine the genetic and physical interactions between four closely-related *A. fumigatus* basic leucine zipper (bZIP) TFs: AtfA, AtfB, AtfC, AtfD. Double deletion mutants were constructed in all pair-wise combinations and phenotypically assessed under stress conditions, revealing intricate interactions of these TF genes during osmotic, oxidative, and cell wall stresses. Furthermore, these TFs physically interact with each other, forming all possible heterodimers. Each TF also physically interacts with the MAP kinase SakA. This work highlights the complexity of TF interactions that mediate response to environment.

Overall, the articles in this Research Topic highlight the regulatory mechanisms controlling fungal TF activity, the genome-wide actions of TFs, interactions between TFs, and the regulation of transcriptional programs in fungi.

## Author contributions

RT wrote the first draft of the manuscript. RT, KW, and GG contributed to manuscript revision, read and approved the submitted version.
